# Calcium supplementation improves clinical outcome in intensive care unit patients: a propensity score matched analysis of a large clinical database MIMIC-II

**DOI:** 10.1186/s40064-015-1387-7

**Published:** 2015-10-13

**Authors:** Zhongheng Zhang, Kun Chen, Hongying Ni

**Affiliations:** Department of Critical Care Medicine, Jinhua Municipal Central Hospital, Jinhua Hospital of Zhejiang University, 351#, Mingyue Road, Jinhua, Zhejiang 321000 China

**Keywords:** Calcium supplementation, Intensive care unit, Mortality, Critically ill

## Abstract

Observational studies have linked hypocalcemia with adverse clinical outcome in critically ill patients. However, calcium supplementation has never been formally investigated for its beneficial effect in critically ill patients. To investigate whether calcium supplementation can improve 28-day survival in adult critically ill patients. Secondary analysis of a large clinical database consisting over 30,000 critical ill patients was performed. Multivariable analysis was performed to examine the independent association of calcium supplementation and 28-day morality. Furthermore, propensity score matching technique was employed to investigate the role of calcium supplementation in improving survival. Intervention: none. Primary outcome was the 28-day mortality. 90-day mortality was used as secondary outcome. A total of 32,551 adult patients, including 28,062 survivors and 4489 non-survivors (28-day mortality rate: 13.8 %) were included. Calcium supplementation was independently associated with improved 28-day mortality after adjusting for confounding variables (hazard ratio: 0.51; 95 % CI 0.47–0.56). Propensity score matching was performed and the after-matching cohort showed well balanced covariates. The results showed that calcium supplementation was associated with improved 28- and 90-day mortality (p < 0.05 for both Log-rank test). In adult critically ill patients, calcium supplementation during their ICU stay improved 28-day survival. This finding supports the use of calcium supplementation in critically ill patients.

## Background

Electrolyte disturbance is commonly seen in critically ill patients as part of the systemic illness. It is estimated that up to 90 % of critically ill patients present disturbance in serum electrolytes, including hypo- and hyperkalemia, hyper- and hyponatremia (Rosner and Ronco 2010; Lee 2010). Calcium is one of the most important ions that plays an important role in maintaining biological homeostasis. The active form of calcium is the ionized calcium (iCa) that can diffuse across cellular membrane and is tightly regulated at both cellular and systemic levels (Wakai and Fissore 2013; Suzuki and Inoue 2010). Disturbances of iCa, including both hypocalcemia and hypercalcemia, are prevalent among ICU patients. Since hypocalcemia is much more prevalent than hypercalcemia in ICU patients, the former is the focus of research interest. Many observational studies have shown that hypocalcemia is associated with adverse outcomes in ICU patients (Steele et al. 2013; Dias et al. 2013; Anastasopoulos et al. 2011). Steele et al. demonstrated that severely hypocalcemic patients required critical care for longer.

However, it is still largely unknown whether hypocalcemia is the cause of poor prognosis or is only a byproduct of the severe illness. If the former is true, there comes the hypothesis that supplementation of calcium for critically ill patients will improve patient’s outcome. Few studies have been conducted to investigate the causal relationship of calcium supplementation and clinical outcomes and the results are conflicting. In a recent experimental study conducted in murine model of sepsis, Collage et al. (2013) found that calcium administration worsened mortality and organ dysfunction, which was mediated via calcium/calmodulin-dependent protein kinase pathway. To the best of our knowledge, there is no direct evidence from human studies demonstrating the causal association of iCa and mortality (Forsythe et al. 2008). Therefore, the present study aimed to investigate the causal relationship between calcium administration and mortality. We hypothesized that calcium administration may potentially benefit ICU patients.

## Methods

### Description of the database

Multiparameter Intelligent Monitoring in Intensive Care II (MIMIC-II, version 2.6) is an open access publicly available ICU database. It consists of more than 30,000 ICU patients (medical, surgical, coronary care and neonatal) admitted to Beth Israel Deaconess Medical Center (Boston, MA, USA) from 2001 to 2008. MIMIC-II is comprised of two major components: clinical data and physiological waveforms. In the present study we employed the clinical data, which included patient demographics, intravenous medication drip rates, and laboratory test results (Lee et al. 2011). The establishment of MIMIC-II clinical database was approved by the Institutional Review Boards of the Massachusetts Institute of Technology (Cambridge, MA, USA) and Beth Israel Deaconess Medical Center (Boston, MA, USA). Our access to the database was approved after completion of the NIH web-based training course named “Protecting Human Research Participants” by the author ZZ. (Certification Number: 1132877). Informed consent was waived due to observational nature of the study. The study was approved by the ethics committee of Jinhua municipal central hospital. Data extraction was performed by using structure query language (SQL) with pgADmin PostgreSQL tools (version 1.12.3) (Scott et al. 2013). MIMIC-II was organized into a relational database that consisted of 38 tables. Data were extracted from the following tables: POE_MED, POE_ORDER, COMORBIDITY_SCORES, ICUSTAY_DETAIL, LABEVENTS.

### Study population and definitions

MIMIC-II consisted both pediatric and adult populations, and only adult patients (>15 years old) were enrolled into current analysis. Patients who had undergone renal replacement therapy were excluded because such treatment had significant impact on serum calcium. Data on following information were extracted: age on ICU admission, sex, comorbidities (including chronic pulmonary disease, congestive heart failure, paralysis, renal failure, liver disease, diabetes, hypertension, alcohol abuse and acquired immunodeficiency syndrome [AIDS]), ethnicity (white, Asian, Black, Hispanic/latino and unknown), serum creatinine on ICU entry, day 1 sequential organ failure assessment (SOFA) and Simplified Acute Physiology Score (SAPS-1), time of ICU admission and discharge, date of death, all measurements of iCa during ICU stay.

Calcium could be administered in the following formula (data obtained from the table POE_MED): calcium, glucose calcium, calcium carbonate, calcium chloride, calcium chloride oral, calcium gluconate, fosamprenavir calcium and rosuvastatin calcium. Among them calcium gluconate and calcium carbonate were the major source of calcium supplementation, accounting for 75 and 13 %, respectively, of the total calcium administration.

The primary endpoint in our study was the 28-day mortality which was defined as death observed within 28 days after ICU entry. Secondary endpoint was 90-day mortality. Length of stay (LOS) in ICU and hospital were also assessed.

### Statistical analysis

Kolmogorov–Smirnov test was used to test the normality of the distribution of continuous variables. Data of normal distribution were expressed as mean ± SD and compared using t test. Otherwise, Wilcoxon rank-sum test was used for comparison. Categorical variables were expressed as percentage and statistical inference was made based on Chi square test or Fisher’s exact test as appropriate. Variables were compared between survivors and non-survivors with univariate analysis to determine screen variables associated with 28-day mortality. Covariates with p < 0.1 in univariate analysis were entered into Cox proportional hazard regression model to determine whether calcium supplementation was independently associated with 28-day mortality.

Covariates presumed to influence the choice of calcium supplementation were included in a multivariable regression model with calcium supplementation as the dependent variable to determine the propensity score of calcium supplementation for each patient (the probability of receiving calcium supplementation conditionally on the observed covariates) (D’Agostino 1998). Independent covariates included in calculating propensity score were sex, SAPSI-1, hypertension, AIDS, renal failure, congestive heart failure, chronic pulmonary disease, iCa on ICU entry. The use of propensity score analysis aimed to reduce the imbalance between two matched cohorts. Radius matching was employed. Standardized differences before and after matching were plotted to show the effect of matching.

In the matched cohort, survival analysis with log-rank test was performed to determine whether calcium supplementation affects 28- and 90-day mortality. Kaplan–Meier survival curve was depicted.

All statistical analyses were performed using the software STATA 11.2 (College Station, TX 77845, USA) and R software. Two-tailed p < 0.05 was considered to be statistically significant.

## Results

Data on 32,551 adult patients were included in our analysis. There were 28,062 survivors and 4489 non-survivors (28-day mortality rate: 13.8 %). As shown in Table 1, variables including age (62.6 ± 17.9 vs. 71.3 ± 16.0, p < 0.001), sex (male percentage: 56.70 % vs. 53.68 %, p < 0.001), SAPS-1 (13.1 ± 5.2 vs. 17.6 ± 5.8, p < 0.001), SOFA (5.1 ± 3.8 vs. 8.1 ± 4.6, p < 0.001), Asian population (3.54 vs. 4.15 %, p = 0.044), hypertension (31.79 vs. 29.18 %, p = 0.001), congestive heart failure (19.88 vs. 31.98 % p < 0.001), chronic pulmonary disease (16.89 % vs. 19.11 %, p < 0.001), renal failure (6.13 vs. 9.71 %, p < 0.001), liver disease (5.06 vs. 7.19 %, p < 0.001), alcohol abuse (5.41 vs. 3.80 %, p < 0.001), serum iCa on ICU entry (1.133 ± 0.103 vs. 1.113 ± 0.146 mmol/L, p < 0.001), serum creatinine (1.32 ± 1.48 vs. 1.76 ± 1.52 mg/dL, p < 0.001) and calcium supplementation (33.56 vs. 31.03 %, p = 0.001) were significantly different between survivors and non-survivors. Variables with p < 0.1 were entered into Cox regression model (Table 2), which showed that calcium supplementation was associated with reduced risk of death (hazard ratio: 0.51; 95 % CI 0.47–0.56).

**Table 1:** Differences of clinical characteristics between survivors and non-survivors (28-day mortality)

Clinical parameters	Total (n = 32,551)	Survivors (n = 28,062)	Non-survivors (n = 4489)	P value
Age (years)	63.8 ± 17.9	62.6 ± 17.9	71.3 ± 16.0	<0.001
Sex (male, %)	18,089 (56.27)	15,682 (56.70)	2407 (53.68)	<0.001
SAPSI-1	13.7 ± 5.5	13.1 ± 5.2	17.6 ± 5.8	<0.001
SOFA	5.5 ± 4.0	5.1 ± 3.8	8.1 ± 4.6	<0.001
Ethnicity (n, %)				
White	21,704 (67.5)	18,684 (67.53)	3020 (67.34)	0.810
Asian	1166 (3.63)	980 (3.54)	186 (4.15)	0.044
Black	2912 (9.06)	2508 (9.06)	404 (9.01)	0.902
Unknown	5261 (16.4)	4526 (16.36)	737 (16.43)	0.901
Hispanic/latino	1108 (3.45)	970 (3.51)	138 (3.08)	0.144
Comorbidities (n, %)				
Hypertension	9200 (31.4)	8064 (31.79)	1136 (29.18)	0.001
Congestive heart failure	6288 (21.49)	5043 (19.88)	1245 (31.98)	<0.001
Chronic pulmonary disease	5029 (17.19)	4285 (16.89)	744 (19.11)	<0.001
Paralysis	398 (1.36)	341 (1.34)	57 (1.46)	0.547
Renal failure	1933 (6.61)	1555 (6.13)	378 (9.71)	<0.001
Liver disease	1563 (5.34)	1283 (5.06)	280 (7.19)	<0.001
Diabetes	1637 (5.59)	1438 (5.67)	199 (5.11)	0.159
Alcohol abuse	1520 (5.19)	1372 (5.41)	148 (3.80)	<0.001
AIDS	208 (0.71)	188 (0.74)	20 (0.51)	0.116
Serum iCa on ICU entry (mmol/L)	1.130 ± 0.111	1.133 ± 0.103	1.113 ± 0.146	<0.001
Serum creatinin on ICU entry (mg/dL)	1.38 ± 1.49	1.32 ± 1.48	1.76 ± 1.52	<0.001
Calcium supplementation (n, %)	10,810 (33.21)	9417 (33.56)	1393 (31.03)	0.001

**Table 2:** Cox regression model showing variables associated with 28-day mortality

Variables	Hazard ratio	Standard error	P value	Lower limit (95 % CI)	Upper limit (95 % CI)
Calcium supplementation	0.509913	0.023411	<0.001	0.466033	0.557925
Sex	0.838076	0.038628	<0.001	0.765685	0.917312
Age	1.020377	0.001796	<0.001	1.016864	1.023903
SAPSI-1 score	1.093642	0.006636	<0.001	1.080713	1.106726
Sofa score	1.074119	0.008373	<0.001	1.057834	1.090655
Ca^2+^ on ICU entry	0.201529	0.040239	<0.001	0.136263	0.298054
Asian population	1.130669	0.124779	0.266	0.910748	1.403695
Congestive heart failure	1.266334	0.064469	<0.001	1.146076	1.399211
Hypertension	1.041436	0.052815	0.423	0.942899	1.150271
Chronic pulmonary disease	1.081551	0.062353	0.174	0.965993	1.210932
Renal failure	0.7697	0.074854	0.007	0.636123	0.931326
Liver disease	1.600751	0.138914	<0.001	1.350381	1.897541
Alcohol abuse	1.258592	0.140858	0.04	1.010697	1.56729
Serum creatinine on ICU entry	1.114954	0.016701	<0.001	1.082696	1.148173

**Fig. 1:**
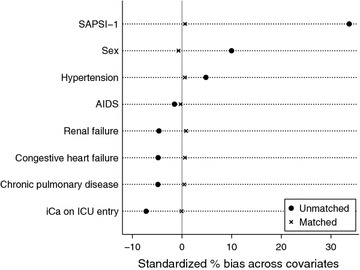
Standardized bias (%) across covariates before and after propensity score matching. The result showed that candidate covariates were well matched

**Fig. 2:**
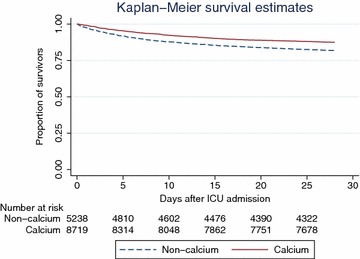
Kaplan–Meier survival curves showing that 28-day mortality was reduced with calcium supplementation

**Fig. 3:**
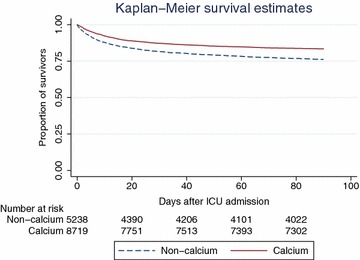
Kaplan–Meier survival curves showing that 90-day mortality was reduced with calcium supplementation

Variables were compared between calcium and non-calcium groups (Table 3). Mild forms of acute renal failure as reflected by mild creatinine elevation was not significantly different between calcium and non-calcium groups (1.36 ± 1.53 vs. 1.38 ± 1.47 mg/dL, p = 0.26). Calcium supplementation was used as the dependent variable. Covariates including sex (59.44 vs. 54.70 %, p < 0.001), SAPS-1 (16.0 ± 5.1 vs. 12.2 ± 5.2, p < 0.001), SOFA (7.5 ± 3.9 vs. 4.4 ± 3.6, p < 0.001), hypertension (30.7 vs. 31.9 %, p = 0.033), congestive heart failure (20.10 vs. 22.28 %, p < 0.001), chronic pulmonary disease (15.92 vs. 17.91 %, p < 0.001), renal failure (6.17 vs. 6.86 %, p = 0.023), AIDS (0.58 vs. 0.78 %, p = 0.047), iCa on ICU entry (1.127 ± 0.104 vs. 1.135 ± 0.121, p < 0.001) were associated with the choice of calcium supplementation. Because SAPSI-1 and SOFA both measured the same clinical characteristics, we used SAPSI-1 to calculate propensity score. After radius matching, there were 5238 in non-calcium group and 8719 in the calcium group. The balance of covariates is shown in Fig. 1, which demonstrates that these variables are well balanced after matching. Figures 2 and 3 are Kaplan–Meier survival curves showing the 28- and 90-day mortality by calcium groups. The results showed that calcium supplementation was associated with improved 28- and 90-day mortality (p < 0.05 for both Log-rank test).

**Table 3:** Differences of clinical characteristics between calcium and non-calcium groups

Clinical parameters	Total (n = 32,551)	Calcium (n = 10,810)	Non-calcium (n = 21,741)	P value
Age (years)	63.8 ± 17.9	63.8 ± 16.9	63.8 ± 18.3	0.98
Sex (male, %)	18,089 (56.27)	6351 (59.44)	11,738 (54.70)	<0.001
SAPS-1	13.7 ± 5.5	16.0 ± 5.1	12.2 ± 5.2	<0.001
SOFA	5.5 ± 4.0	7.5 ± 3.9	4.4 ± 3.6	<0.001
Ethnicity (n, %)				
White	21,704 (67.5)	7260 (67.96)	14,444 (67.3)	0.212
Asian	1166 (3.63)	393 (3.68)	773 (3.60)	0.722
Black	2912 (9.06)	936 (8.76)	1976 (9.20)	0.195
Unknown	5261 (16.4)	1736 (16.3)	3527 (16.4)	0.689
Hispanic/latino	1108 (3.45)	357 (3.34)	751 (3.50)	0.471
Comorbidities (n, %)				
Hypertension	9200 (31.4)	3267 (30.7)	5933 (31.9)	0.033
Congestive heart failure	6288 (21.49)	2141 (20.10)	4147 (22.28)	<0.001
Chronic pulmonary disease	5029 (17.19)	1696 (15.92)	3333 (17.91)	<0.001
Paralysis	398 (1.36)	140 (1.31)	258 (1.39)	0.610
Renal failure	1933 (6.61)	657 (6.17)	1276 (6.86)	0.023
Liver disease	1563 (5.34)	553 (5.19)	1010 (5.43)	0.390
Diabetes	1637 (5.59)	565 (5.30)	1072 (5.76)	0.103
Alcohol abuse	1520 (5.19)	545 (5.12)	975 (5.24)	0.651
AIDS	208 (0.71)	62 (0.58)	146 (0.78)	0.047
Serum iCa on ICU entry (mmol/L)	1.130 ± 0.111	1.127 ± 0.104	1.135 ± 0.121	<0.001
Serum creatinine on ICU entry (mg/dL)	1.38 ± 1.49	1.36 ± 1.53	1.38 ± 1.47	0.26
Outcomes				
28-day mortality (n, %)	4489 (13.79)	1393 (12.89)	3096 (14.24)	0.001
90-day mortality (n, %)	6000 (18.43)	1846 (17.08)	4154 (19.11)	<0.001
ICU LOS (days; median IQR)	2.11 (1.11–4.21)	3.25 (1.89–7.04)	1.79 (0.96–3.19)	<0.001
Hospital LOS (days; median; IQR)	7 (4–14)	10 (6–17)	6 (4–12)	<0.001

### Multivariable dose–response relationship

**Fig. 4:**
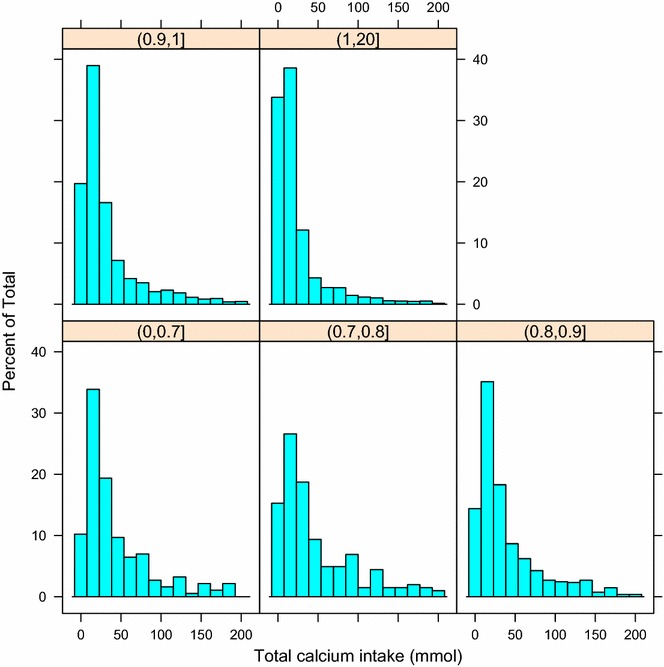
Distribution of total calcium intake stratified by serum iCa. The overall median calcium intake was 13.9 mmol (interquartile range: 4.6–111.9 mmol)

In patients received calcium supplementation, we investigated the dose–response relationship. The median calcium intake was 13.9 mmol (interquartile range: 4.6–111.9 mmol) in patients received calcium supplementation during their ICU stay. Distribution of total calcium intake stratified by different serum iCa is displayed in Fig. 4. The multivariable model included variables age, sex, SAPS-1, SOFA, Asian population, hypertension, congestive heart failure, chronic pulmonary disease, renal failure, liver disease, alcohol abuse, serum iCa on ICU entry and serum creatinine (Table 4). The result showed that the dose of calcium intake was significantly associated with 90-day mortality (hazard ratio: 1.0004, p < 0.001).

**Table 4:** Cox proportional hazard model investigating the association of calcium supplementation with 90-day mortality

	Hazard ratio	95 % lower limit	95 % upper limit	p
Total calcium (mmol)	1.0004	1.0002	1.0007	<0.001
Creatinine on entry	1.1161	1.0892	1.1436	<0.001
First calcium	0.2873	0.1935	0.4267	<0.001
Sex	0.9201	0.8502	0.9958	<0.05
Age	1.0306	1.0274	1.0338	<0.001
SASP-1	1.0333	1.0221	1.0446	<0.001
SOFA	1.0181	1.0040	1.0323	<0.05
Minimum calcium	0.6786	0.4673	0.9855	<0.05
Asian population	1.0105	0.8232	1.2404	>0.05
Hypertension	0.7725	0.7074	0.8437	<0.001
Congestive heart failure	1.6238	1.4891	1.7707	<0.001
Chronic pulmonary disease	1.1956	1.0864	1.3158	<0.001
Renal failure	1.2153	1.0375	1.4235	<0.05
Liver disease	1.8596	1.5968	2.1656	<0.001
Alcohol abuse	1.2424	1.0165	1.5185	<0.05

### Subgroup analysis

**Fig. 5:**
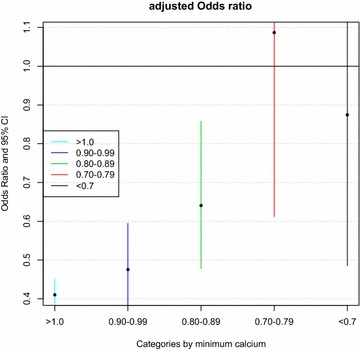
Subgroup analysis by dividing patients into subsets according to their minimum iCa. Within each subgroup, multivariable regression model was used to control for confounding factors including creatinine, sex, age, SAPS-I, SOFA, Asian population, hypertension, congestive heart failure, chronic pulmonary disease, renal failure, liver disease and alcohol abuse

Patients were grouped into subsets by their minimum calcium levels of >1.0 (11,404), 0.9–0.99 (2388), 0.8–0.89 (1326), 0.7–0.79 (343) and <0.7 (316). Multivariable regression model was fitted to adjust for confounding variables to investigate the effect of calcium supplementation on mortality risk. The results showed that in subgroups with iCa >1, calcium supplementation was associated with reduced risk of death (OR: 0.41, 95 % CI 0.37–0.45). Results of other subgroups are shown in Fig. 5.

## Discussion

Our study for the first time demonstrates that calcium supplementation is beneficial for the critically ill patients. In multivariable regression model, calcium supplementation is an independent protective factor for 28-day mortality. After propensity score matching, patients receiving calcium supplementation showed significantly improved survival time, as compared with those without calcium administration during ICU stay. Ionized calcium plays an important role in maintaining normal physiological function, particularly in signal transduction pathway (Ritchie et al. 2011). Many studies in critical care setting have demonstrated that hypocalcemia was associated with significantly increased risk of death. In patients with acute kidney injury needing renal replacement therapy, Afshinnia et al. (2013) showed that severe hypocalcemia with iCa  < 1  mmol/L was associated with increased risk of death. However, this does not hold true in patients with mild hypocalcemia. Steele et al. (2013) also showed that failure to normalization of ionized calcium in severely hypocalcemic patients might be associated with increased mortality. Since hypocalcemia is independently associated with increased mortality, it is not surprising that calcium supplementation is associated with improved outcome.

There is no direct evidence in the causal relationship between calcium supplementation and mortality in critically ill patients. In an animal study, Collage et al. (2013) demonstrated that calcium supplementation mediated heightened inflammation and vascular leak, culminating in elevated organ dysfunction and mortality. Furthermore, they found that this negative impact of calcium supplementation was associated with calcium/calmodulin-dependent protein kinase kinase signaling. This finding is in contrast with our findings. However, our limited understanding on the physiology of calcium cannot fully account for the disparity between animal studies and our clinical observation.

The strength of the present study is the use of a large clinical database MIMIC-II. This is an open access database comprising clinical data of more than 30,000 ICU patients. As compared to well-designed randomized controlled trial, the secondary analysis of such “real-world” data may be more generalizable. RCTs have been criticized for its complexity (e.g. strict inclusion/exclusion criteria, strict study protocol), requirement of specialized center (RCT requires authorized center with special facilities), homogeneity of included subjects, and the unrepresentative of usual care (e.g. early goal directed therapy may not be strictly implemented in real world setting as described in study protocol), making the result of RCTs less generalizable to the “real world” setting (Albert et al. 2013). However, observational study is subject to confounding bias. In the present study, we employed a multi-variable regression model and propensity score matching techniques to control for confounding factors. Both methods consistently showed that calcium supplementation was able to improve clinical outcomes.

Several limitations need to be acknowledged in our study. One limitation is the exclusion of patients undergoing renal replacement therapy, and the interaction between calcium supplementation and renal function was not investigated. It would be interesting to examine how calcium supplementation will impact renal function. However, this study is based on database analysis. The database is collected from routine clinical practices. There is no standard protocol on the timing of the measurements of calcium and renal function. Thus, missing data and sequence of measurement of creatinine and calcium prohibit further analysis. Calcium supplementation may have different effect on different disease entities and indications. There are varieties of conditions that can cause hypocalciumia. Common causes include hypocalcemia with high parathyroid hormone (PTH), chronic kidney disease, vitamin D deficiency, hypocalcemia with low PTH, hypoparathyroidism, deregulation of PTH, acute pancreatitis, severe hypo-magnesemia due to suppression of PTH release, sepsis or severe illness. However, the indication for calcium supplementation cannot be extracted from database analysis. Ideally, the dose of calcium supplementation would have been investigated to determine the dose–response relationship. However, the doses of calcium are also difficult to determine due to differences in calcium formula, route of administration and duration. Due to these limitations, the interpretation of the result should be cautious, and further experimental trials are mandatory to confirm or refute our findings. In current analysis we identify a dose–response relationship in calcium supplementation and mortality, but the result was unexpected in that more calcium intake was associated with increased risk of death. In subgroup analysis, calcium supplementation appeared to have no beneficial effect in patients with severe hypocalcemia. Collectively, these results suggest that calcium intake is not “the more, the better”. Dose–response analysis was performed in patients receiving calcium during their ICU stay. Although we have controlled for minimum serum iCa, it cannot be excluded that severe hypocalcemia is a biomarker of disease severity. Furthermore, total calcium intake calculated in our study is heterogeneous that they differ in formula, administration route, frequency, dose and duration. As this is a study of data mining, it is impossible to standardize these details. Another reason might be that the sample size in patients with severe hypocalcemia was small after stratification, which limited the statistical power (wide confidence interval) and made the random error become large. Thus, it is mandatory to conduct a randomized controlled trial to test whether calcium intake is associated with improved outcome.

In aggregate, our study for the first time suggests that calcium supplementation may be helpful in reducing mortality in critically ill patients.
